# The role of immune suppression in COVID-19 hospitalization: clinical and epidemiological trends over three years of SARS-CoV-2 epidemic

**DOI:** 10.3389/fmed.2023.1260950

**Published:** 2023-09-07

**Authors:** Marta Canuti, Maria Cristina Monti, Chiara Bobbio, Antonio Muscatello, Toussaint Muheberimana, Sante Leandro Baldi, Francesco Blasi, Ciro Canetta, Giorgio Costantino, Alessandro Nobili, Flora Peyvandi, Mauro Tettamanti, Simone Villa, Stefano Aliberti, Mario C. Raviglione, Andrea Gori, Alessandra Bandera, COVID-19 Network Study Group, Bosari Silvano, Scudeller Luigia, Fusetti Giuliana, Rusconi Laura, Dell’Orto Silvia, Prati Daniele, Valenti Luca, Giovannelli Silvia, Manunta Maria, Lamorte Giuseppe, Ferarri Francesca, Mangioni Davide, Alagna Laura, Bozzi Giorgio, Lombardi Andrea, Ungaro Riccardo, Ancona Giuseppe, Zuglian Gianluca, Bolis Matteo, Iannotti Nathalie, Ludovisi Serena, Comelli Agnese, Renisi Giulia, Biscarini Simona, Castelli Valeria, Palomba Emanuele, Fava Marco, Fortina Valeria, Liparoti Arianna, Pastena Andrea, Alberto Peri Carlo, Saltini Paola, Viero Giulia, Itri Teresa, Ferroni Valentina, Pastore Valeria, Massafra Roberta, Curri Maria Teresa, Rizzo Alice, Scarpa Stefano, Giommi Alessandro, Bianco Rosaria, Chitani Grazia Eliana, Gualtierotti Roberta, Ferrari Barbara, Rossio Raffaella, Boasi Nadia, Pagliaro Erica, Massimo Costanza, Caro Michele De, Giachi Andrea, Montano Nicola, Vigone Barbara, Bellocchi Chiara, Carandina Angelica, Fiorelli Elisa, Melli Valerie, Tobaldini Eleonora, Spotti Maura, Terranova Leonardo, Misuraca Sofia, D’Adda Alice, Fiore Silvia Della, Pasquale Marta Di, Mantero Marco, Contarini Martina, Ori Margherita, Morlacchi Letizia, Rossetti Valeria, Gramegna Andrea, Pappalettera Maria, Cavallini Mirta, Buscemi Agata, Vicenzi Marco, Rota Irena, Solbiati Monica, Furlan Ludovico, Mancarella Marta, Colombo Giulia, Colombo Giorgio, Fanin Alice, Passarella Mariele, Monzani Valter, Rovellini Angelo, Barbetta Laura, Billi Filippo, Folli Christian, Accordino Silvia, Maira Diletta, Hu Cinzia Maria, Motta Irene, Scaramellini Natalia, Fracanzani Anna Ludovica, Lombardi Rosa, Cespiati Annalisa, Cesari Matteo, Lucchi Tiziano, Proietti Marco, Calcaterra Laura, Mandelli Clara, Coppola Carlotta, Cerizza Arturo, Grasselli Giacomo, Galazzi Alessandro, Monti Igor, Galbusera Alessia Antonella

**Affiliations:** ^1^Department of Pathophysiology and Transplantation, Università degli Studi di Milano, Milan, Italy; ^2^Centre for Multidisciplinary Research in Health Science (MACH), Università degli Studi di Milano, Milan, Italy; ^3^Coordinate Research Centre EpiSoMI (Epidemiology and Molecular Surveillance of Infections), Università degli Studi di Milano, Milan, Italy; ^4^Department of Public Health, Experimental and Forensic Medicine, Unit of Biostatistics and Clinical Epidemiology, Università degli Studi di Pavia, Pavia, Italy; ^5^Fondazione IRCCS Ca’ Granda Ospedale Maggiore Policlinico di Milano, Milan, Italy; ^6^Department of Clinical Sciences and Community Health, Università degli Studi di Milano, Milan, Italy; ^7^Department of Health Policy, Istituto di Ricerche Farmacologiche Mario Negri IRCCS, Milan, Italy; ^8^Department of Biomedical Sciences, Humanitas University, Milan, Italy; ^9^IRCCS Humanitas Research Hospital, Respiratory Unit, Milan, Italy

**Keywords:** SARS-CoV-2, COVID-19, disease outcome, hospitalization, COVID-19 vaccination, immune suppression

## Abstract

Specific immune suppression types have been associated with a greater risk of severe COVID-19 disease and death. We analyzed data from patients >17 years that were hospitalized for COVID-19 at the “Fondazione IRCCS Ca′ Granda Ospedale Maggiore Policlinico” in Milan (Lombardy, Northern Italy). The study included 1727 SARS-CoV-2-positive patients (1,131 males, median age of 65 years) hospitalized between February 2020 and November 2022. Of these, 321 (18.6%, CI: 16.8–20.4%) had at least one condition defining immune suppression. Immune suppressed subjects were more likely to have other co-morbidities (80.4% vs. 69.8%, *p* < 0.001) and be vaccinated (37% vs. 12.7%, *p* < 0.001). We evaluated the contribution of immune suppression to hospitalization during the various stages of the epidemic and investigated whether immune suppression contributed to severe outcomes and death, also considering the vaccination status of the patients. The proportion of immune suppressed patients among all hospitalizations (initially stable at <20%) started to increase around December 2021, and remained high (30–50%). This change coincided with an increase in the proportions of older patients and patients with co-morbidities and with a decrease in the proportion of patients with severe outcomes. Vaccinated patients showed a lower proportion of severe outcomes; among non-vaccinated patients, severe outcomes were more common in immune suppressed individuals. Immune suppression was a significant predictor of severe outcomes, after adjusting for age, sex, co-morbidities, period of hospitalization, and vaccination status (OR: 1.64; 95% CI: 1.23–2.19), while vaccination was a protective factor (OR: 0.31; 95% IC: 0.20–0.47). However, after November 2021, differences in disease outcomes between vaccinated and non-vaccinated groups (for both immune suppressed and immune competent subjects) disappeared. Since December 2021, the spread of the less virulent Omicron variant and an overall higher level of induced and/or natural immunity likely contributed to the observed shift in hospitalized patient characteristics. Nonetheless, vaccination against SARS-CoV-2, likely in combination with naturally acquired immunity, effectively reduced severe outcomes in both immune competent (73.9% vs. 48.2%, *p* < 0.001) and immune suppressed (66.4% vs. 35.2%, *p* < 0.001) patients, confirming previous observations about the value of the vaccine in preventing serious disease.

## 1. Introduction

SARS-CoV-2 (severe acute respiratory syndrome coronavirus 2) infections have highly variable outcomes in different patients, with a clinical spectrum varying from entirely asymptomatic to respiratory failure, septic shock, multiple organ dysfunction, and death (1). Older age and several co-morbidities have been identified to be unequivocally associated with worse COVID-19 (coronavirus disease 2019) outcomes (2). The presence of one or more co-morbidities (multimorbidity) can exacerbate pathological mechanisms occurring during the infection and/or reduce the tolerance of the patient to organ injury (3). For example, chronic kidney, lung, or liver diseases, diabetes, cardiovascular disease, obesity, and cancer have all been associated with an increased risk of progressing to severe COVID-19. Given this variability, the individual immune response to SARS-CoV-2 is likely also affecting the clinical course of the disease (2, 3).

In literature, contradictory opinions about whether immune suppression is a significant risk factor for COVID-19 exist. On one hand, COVID-19 incidence, morbidity, and mortality rates do not seem to differ largely between immune suppressed individuals and the general population, and immune suppressed patients seem to present more favorable outcomes as compared to patients with other types of co-morbidities (3–5) not directly associated with immune suppression. On the other hand, patients with specific types of immune suppression, like those linked to human immunodeficiency virus (HIV) infection, solid organ transplantation, or B-cell depleting therapies, have a greater risk for severe COVID-19 outcomes, such as those requiring ventilation or extracorporeal membrane oxygenation (ECMO), and death (3, 6). Indeed, several factors can influence the immune status of an individual, and immune suppression can have different causes, including genetic disorders, tumors, infections, or pharmacological treatments, and our understanding of COVID-19 clinical outcomes associated with different types of immune suppression is limited. Furthermore, determining the outcome severity in immune suppressed individuals may be complicated as several factors, such as a disease, its treatment, or a disease-related immune suppression, can influence the clinical course of an infection (7).

Another aspect to consider is that a state of immune suppression may reduce the response to vaccine-induced immunizations and subjects with immune dysfunctions may be at higher risk for contracting a breakthrough infection (6). Additionally, studies suggest that some immune suppressed patients, especially those with immune-mediated inflammatory diseases and those on B cell-depleting therapies, remain susceptible to poor outcomes even after vaccination (8, 9). Therefore, when a high vaccination coverage has been achieved, patients with immune dysfunctions may represent a substantial proportion of hospitalized and deceased patients.

In this study, we analyzed clinical data collected from patients hospitalized for COVID-19 at the “Fondazione IRCCS Ca′ Granda Ospedale Maggiore Policlinico” in Milan (Lombardy, Northern Italy) since February 2020–when SARS-CoV-2 was first recognized in Italy–until the end of 2022. Lombardy was one of the first non-Asian areas with sustained SARS-CoV-2 transmission, the first epicenter of the European epidemic, and the Italian region with the highest COVID-19 clinical burden in early 2020 (10–12). In fact, in March–May 2020, Lombardy experienced a 111.8% increase in all-cause deaths compared with the same period in the quinquennia 2015–2019 (excess deaths due to all causes), being one of the heaviest contributors to the Italian overall 31.7% increase in excess mortality (10, 13). Afterward, following global trends, cycles of infection peaks and dips occurred in Lombardy as different variants characterized by diverse degrees of transmissibility and pathogenicity spread and became prevalent during different periods (14–16).

The main scope of this retrospective observational study was to evaluate the contribution of immune suppression to hospitalization during the various stages of the COVID-19 epidemic, which were characterized by the circulation of different variants and different degrees of vaccination coverage, by studying a cohort of patients hospitalized for COVID-19 in one Hospital in Milan between the end of February 2020 and November 2022. Additionally, we investigated whether immune suppression contributed to severe outcomes and death and assessed whether vaccination reduced severe outcomes and death also in immune suppressed patients.

## 2. Materials and methods

### 2.1. Data collection

This was an observational cohort study (COVID-19 Network Registry). The study population consisted of patients aged >17 years who were hospitalized at Fondazione IRCCS Ca′ Granda Ospedale Maggiore Policlinico of Milan and who were positive for SARS-CoV-2 based on real-time PCR. Patients that were directly admitted to the intensive care unit (ICU) were excluded. The study was approved by the Medical Ethics Committee of the Fondazione IRCCS Ca′ Granda Ospedale Maggiore Policlinico (EC approval 241_2020, 17 March 2020). The need to obtain informed consent was waived by the Medical Ethics Committee in cases where it was not possible to obtain informed consent, due to severe illness or death. In all other cases, written informed consent was obtained. Ethnicity was retrieved from medical charts. Study data were collected and managed using Research Electronic Data Capture (REDCap^®^) (17).

### 2.2. Study population, inclusion and exclusion criteria, variable definition

The study included 1727 SARS-CoV-2-positive nonminor (>17 years of age) patients that had been admitted to the hospital “Fondazione IRCCS Ca′ Granda Ospedale Maggiore Policlinico,” Milan (Lombardy, Italy), between the end of February 2020 and November 2022. As information about hospitalization in other facilities for transferred patients could not be obtained, we considered only the period between the admission to and discharge from the COVID-19 unit of this hospital and all included patients that remained hospitalized for at least 1 day (distinct dates of admission and discharge). For all patients, sex at birth, ethnicity, and smoking status were recorded. Patients were divided into 3 age classes (18–50 years, 51–70 years, and > 70 years), according to what is routinely done in the epidemiological reports of the Istituto Superiore di Sanita’, the National Health agency monitoring the epidemiology of SARS-CoV-2 in Italy (18).

Information about the immune status of all participants was available and patients were divided into two groups. We considered as immune suppressed (exposure) those patients with at least one of the following conditions: (i) history of any connective tissue disease, autoimmune disease, and/or primary immunodeficiency; (ii) history of an active solid or hematologic tumor; (iii) neutropenia; (iv) diagnosis of HIV or acquired immunodeficiency syndrome (AIDS); (v) history of splenectomy, solid organ transplantation, and/or hematopoietic stem cell transplantation; (vi) ongoing treatment with biological drugs during the six months prior to admission or with corticosteroids, chemotherapy, and/or other immune suppressive agents during the 3 months prior to admission. All other patients were considered immune competent.

In consideration of the fact that patient history also included the presence of co-morbidities other than immune suppression, we divided patients co-morbidities other than the ones considered above into six categories: (i) respiratory diseases: chronic obstructive pulmonary disease (COPD), asthma, interstitial lung disease, bronchiectasis; (ii) cardiovascular diseases: heart failure, atrial fibrillation, myocardial infarction, hypertension, pulmonary hypertension; (iii) nephropathies: chronic kidney disease, dialysis; (iv) gastrointestinal diseases, including gastroesophageal reflux disease, and liver diseases, including liver cirrhosis, hepatitis C virus (HCV) infection, and the presence of markers for hepatitis B virus (HBV) infection (HBsAg, HBcAb, HBsAb); (v) metabolic diseases: diabetes, dyslipidemia, malnutrition, and obesity; (vi) neurologic diseases: stroke, transient ischemic attack, and dementia.

Patient vaccination status was also considered. Since complete information (number of doses and dates of administration) about vaccination was available only for a small subset of subjects, patients were considered vaccinated if they received at least one dose of a COVID-19 vaccine (any brand) prior to hospital admission or non-vaccinated if they were hospitalized prior to March 2021 (when the first vaccine doses were made available in Italy) or, for later periods, if they declared not to have been vaccinated against SARS-CoV-2.

We considered a COVID-19 severe outcome when a patient suffered from pneumonia, acute respiratory distress syndrome (ARDS), septic shock, was admitted to an intensive care unit (ICU), or was subjected to mechanical ventilation (intubation). Death during hospitalization caused by SARS-CoV-2 as a main factor or as a co-factor was also considered a severe outcome. All other outcomes were considered favorable outcomes.

### 2.3. Statistical analyses

Continuous measurements were expressed as medians and compared using the permutation-based Mann–Whitney test. Categorical variables were expressed in percentages or proportions and were compared using the Chi-square test or, in case of a small sample size and where appropriate, Fisher’s exact test. Confidence on observed proportions is expressed as 95% normal intervals while for medians the 25th and 75th percentiles are indicated (inter quartile range–IQR). Simple and multiple logistic regression models were used to estimate severe outcome risk factors; odds ratios (OR) with relative 95% intervals of confidence (95% IC) were considered as the measure of effect and precision, respectively. Potential predictors were age and number of co-morbidities (1, 2, 3, >3)–included as continuous variables–and sex, immune status, vaccination, and period (before and since December 2021) – included as nominal variables. The Wald test was used to assess the significance of the regression beta coefficients. Two-sided *p*-values <0.05 were considered statistically significant and Bonferroni correction was applied as appropriate and where indicated. A network analysis was performed with Past using the Rho similarity index with an edge cut-off of 5%. The clustering analysis was also performed in Past using the neighbor-joining algorithm with 1,000 bootstrap resamplings to assess branch robustness.

Analyses were conducted using Past 4.08 (19) and JASP 0.17.1 (20). Final image editing was performed with Inkscape (21).

## 3. Results

### 3.1. Population description

The investigated population of 1727 hospitalized subjects included 1,131 (65.5%) males and 596 females with a median age of 65 (range: 19–100) years. Patient characteristics are summarized in Table 1. Among all considered patients, 1,406 (81.4%, CI: 79.6–83.2%) were immune competent, while 321 (18.6%, CI: 16.8–20.4%) presented one or more factors of immune suppression. As shown in Table 2, the most frequent immune suppression condition was the presence of active cancer (169/321, 52.6%), with an equal presence of solid and hematologic cancers (*p* = 0.5). Drug-induced immune suppression (168/321, 52.3%), including biological drugs, chemotherapy, corticosteroids, or other drugs, was the second most frequent condition, followed by connective tissue disease (17.4%) and organ transplant (16.5%). The proportions of all other conditions were below 10%. A network and a clustering analysis of factors of immune suppression for the studied population are shown in Supplementary Figure S1.

**Table 1:** Characteristics of the studied population and sub-populations.

		Total		Immune competent		Immune suppressed		*p* ^1^
		*N*	%	*N*	%	*N*	%	
	Total	1,727	–	1,406	81.4	321	18.6	
Sex	Males	1,131	65.5	949	67.5	182	56.7	**< 0.001**
	Females	596	34.5	457	32.5	139	43.3	
Age	18–50 years	335	19.4	287	20.4	48	15.0	**0.038**
	51–70 years	723	41.9	590	42.0	133	41.4	
	>70 years	668	38.7	528	37.6	140	43.6	
Ethnicity	Caucasian	1,301	88.1	1,039	87.2	262	92.3	0.20
	Hispanic	77	5.2	68	5.7	9	3.2	
	Asian	49	3.3	42	3.5	7	2.5	
	Arab	29	2.0	27	2.3	2	0.7	
	African descent	12	0.8	10	0.8	2	0.7	
	Other	8	0.5	6	0.5	2	0.7	
Smoker	No	806	71.8	666	72.9	140	67.3	0.18
	Past	233	20.8	180	19.7	53	25.5	
	Current	83	7.4	68	7.4	15	7.2	
Co-morbidities^2^	None	424	24.6	424	30.2	63	19.6	**< 0.001**
	At least one	1,303	75.4	982	69.8	258	80.4	
	Cardiovascular diseases	902	52.2	721	51.3	181	56.4	0.098
	Metabolic diseases	628	36.4	503	35.8	125	38.9	0.28
	GI/liver diseases^3^	292	16.9	197	14.0	95	29.6	**< 0.001**
	Respiratory diseases	243	14.1	185	13.2	58	18.1	**0.022**
	Nephropathies	163	9.4	108	7.7	55	17.1	**< 0.001**
	Neurologic diseases	162	9.4	132	9.4	30	9.3	1
	More than 1 category^4^	825	47.8	567	40.3	167	52.0	**< 0.001**
Vaccination^5^	No	1,291	82.8	1,109	87.3	182	63.0	**< 0.001**
	At least 1 dose	268	17.2	161	12.7	107	37.0	

1 *p* values for statistically significant differences calculated between the two groups of immune competent and immune suppressed patients are highlighted in bold.

2 Presence of at least one condition of one of the considered categories and excluding immune suppression for immune suppressed patients.

3 GI, gastrointestinal.

4 Presence of at least one condition of more than one of the considered categories.

5 History of vaccination against SARS-CoV-2 (any brand).

**Table 2:** Conditions causing immune suppression in the 321 immune suppressed patients considered in this study.

Condition	N. patients	Proportion (%)	95% confidence interval
All tumors	169	52.6	47.1–58.1
Solid tumors	85	26.5	21.7–31.3
Hematologic tumors	94	29.3	24.3–34.3
All immune suppressants	168	52.3	46.8–57.8
Biological drugs^1^	53	16.5	12.4–20.6
Chemotherapeutics^2^	69	21.5	17.0-26.0
Corticosteroids and other drugs^2^	92	28.7	23.8–33.7
All connective tissue diseases	56	17.4	13.3–21.6
Rheumatoid arthritis	19	5.9	3.3–8.5
Other connective tissue diseases	38	11.8	8.3–15.3
Transplant recipient	53	16.5	12.4–20.6
Neutropenia	22	6.9	4.1–9.7
HIV/AIDS	18	5.6	3.1–8.1
Asplenia	10	3.1	1.2–5.0
Aplastic anemia	9	2,8	1.0–4.6
Other autoimmune diseases	5	1.6	0.2–3.0
A/Hypogammaglobulinemia	4	1.2	0.0–2.4

1 During the six months prior to admission.

2 During the three months prior to admission.

While ethnicity distribution and smoking habits were similar between immune competent and immune suppressed patients, immune suppressed subjects were slightly older (*p* = 0.038) and the proportion of males was significantly lower among immune suppressed patients (67.5% vs. 56.7%, *p* < 0.001) (Table 1). Additionally, immune suppressed subjects were more likely to have other co-morbidities (*p* < 0.001), and differences were statistically significant for co-morbidities belonging to the categories of gastrointestinal and liver diseases, respiratory diseases, and nephropathies. Finally, the vaccination rate was higher in immune suppressed subjects (37% vs. 12.7%, *p* < 0.001), likely because vaccination was offered earlier to this sub-population.

Comparing different sub-populations of immune suppressed patients to the immune competent population, however, revealed some group-specific differences (Supplementary Table S1). Particularly, patients with cancer and those undergoing chemotherapy, categories tightly connected in the network and clustering analyses (Supplementary Figure S1), were older while transplant recipients and individuals with HIV infection were younger. Additionally, the proportion of females was higher in most sub-groups, but the difference was significant only for cancer patients, those with connective tissue diseases, and individuals taking immune suppressant drugs. Respiratory co-morbidities were more frequent in patients taking corticosteroids and biological drugs; nephropathies were particularly prevalent in transplant recipients and patients taking corticosteroids, which were two tightly connected categories in the correlation analysis; gastrointestinal and hepatic problems were frequent in most immune suppressed groups, except in those with connective tissue diseases.

### 3.2. Temporal trends

**Figure 1:**
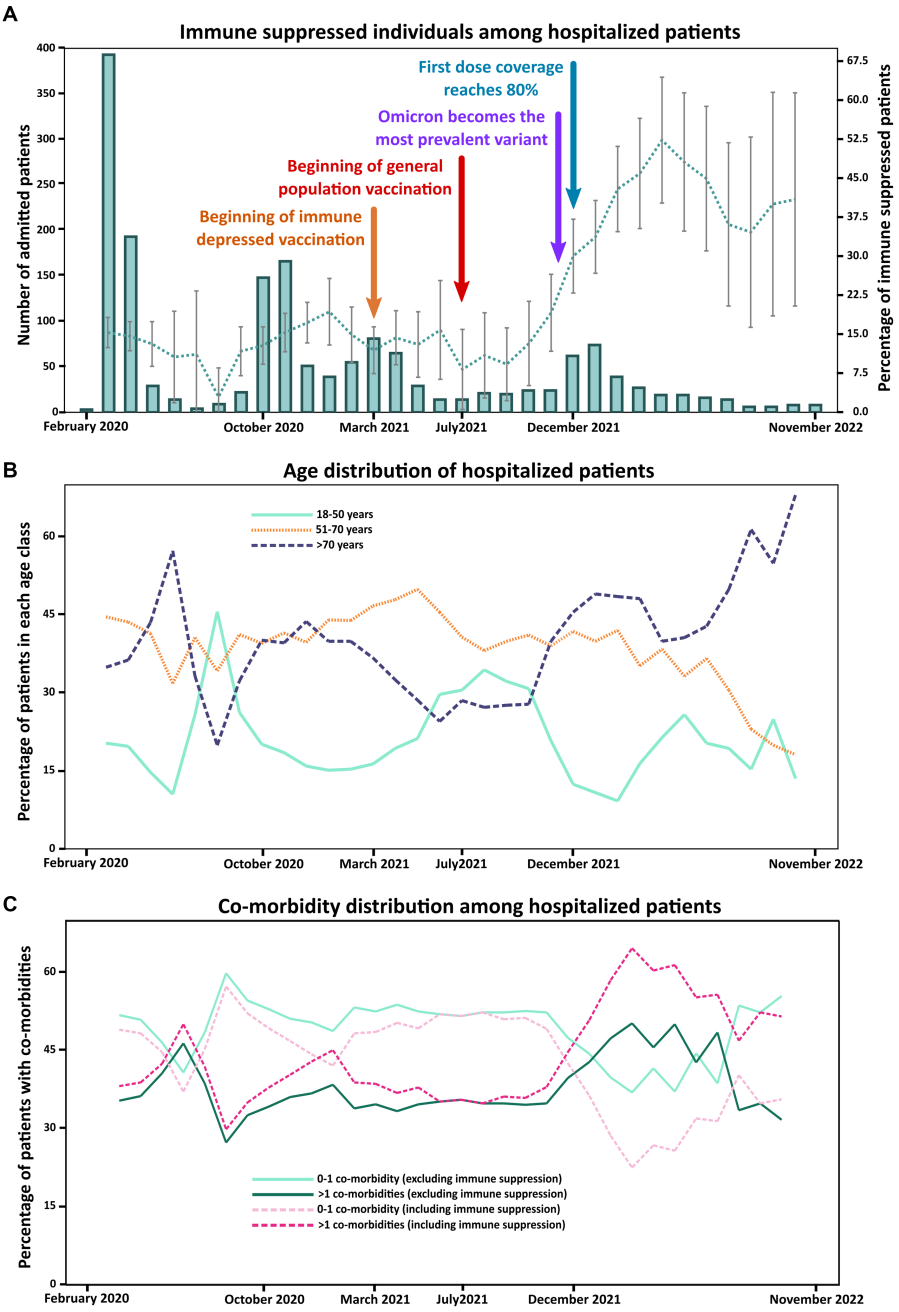
Temporal trends in patient characteristics throughout the study period. The graph in **(A)** represents the proportion of immune suppressed subjects among hospitalized patients. The bar graph (scale on the left) shows the number of hospitalized patients during each month while the dotted line (scale on the right) corresponds to the proportion of immune suppressed patients (for each timepoint the data of three months – the indicated timepoint ± 1 month – were used) with the confidence interval indicated by vertical lines. Timepoints corresponding to key events regarding vaccination or variant circulation are indicated by arrows. The graph in **(B)** shows the relative proportion of patients belonging to the three indicated age classes at the same three-month timepoints. The graph in **(C)** illustrates the proportions of patients hospitalized with no co-morbidities or co-morbidities in one considered category and of subjects with co-morbidities in more than one considered category at the same three-month timepoints; proportions calculated both considering and excluding immune suppression as a co-morbidity category are shown.

To assess whether immune suppression contributed differently to hospitalization during the various phases of the pandemic, we evaluated the proportion of immune suppressed patients among all hospitalizations over time. This proportion remained stable below 20% (mostly between 10 and 20%) until November 2021 but increased to 30% around December 2021 and remained high (30–50%) until the end of the studied period (Figure 1A).

This change coincided with a shift in age distribution as, during the second period, we observed an increase in the proportion of older patients (>70 years: 36.5 vs. 48.7%, *p* < 0.001) and a decrease in the proportion of younger patients (18–50 years: 20.5% vs. 14.4%, *p* = 0.017), while the proportion of patients in the middle age category did not change significantly (51–70: 43.1% vs. 36.9%, *p* = 0.051) (Figure 1B). Older patients presented significantly more immune suppression factors compared to the youngest age group (21.0% vs. 14.3%, *p* = 0.011, with a significance cut-off of 0.025 due to Bonferroni correction) but not compared to the middle age class (18.4%, *p* = 0.23). Nonetheless, similar increasing trends in the proportion of subjects with immune suppression were observed in all three considered age groups (Supplementary Figure S2A) and the proportion of immune suppressed patients in the period from December 2021 until the end was significantly higher than the one in the period from the end of February 2020 to November 2021 in all age groups (*p* < 0.001; Supplementary Table S2).

Similar observations were made for co-morbidities. Around December 2021 the proportion of patients that had co-morbidities in more than one category increased while the proportion of patients with less co-morbidities decreased (Figure 1C). This increase was statistically significant, both considering and excluding immune suppression as a category of co-morbidity (Supplementary Table S3).

In summary, the characteristics of hospitalized patients were very different in these two different periods as patients from December 2021 onwards were older, presented a higher number of co-morbidities, and a higher proportion of them had immune suppression-related factors. In December 2021, Italy reached a COVID-19 first-dose vaccination coverage of 80% (22). The vaccination status of the investigated population is illustrated in Supplementary Figure S3. Additionally, based on national data about variant circulation (15), we observed that December 2021 also corresponded to the moment when the Omicron variants started to become the most prevalent (Supplementary Figure S2B). Overall, we could not definitely conclude whether the noted shifts were due to the reached high immunity coverage, the spread of the Omicron variant, or both.

### 3.3. Infection outcome

Overall, severe outcomes (including death) were observed in 843 patients, and COVID-19-associated deaths were documented in 254 (30.1%) of these individuals. Among immune competent patients, severe outcomes and death were observed in 47.9% (674/1406) and 13.5% (190/1406) of cases, respectively, while they were observed in 52.7% (169/321) and 19.9% (64/321) of immune suppressed subjects, respectively. Only mortality was significantly higher in immune suppressed patients compared to immune competent subjects (*p* = 0.003). Similarly, considering the various conditions of immune suppression separately, a severe outcome was recorded significantly more frequently, compared to immune competent subjects, only among patients treated with biological drugs and patients with connective tissue diseases (Table 3).

**Table 3:** Patients with severe outcomes stratified by type of immune suppression and period of hospitalization.

Status/Condition	Patients with severe outcomes					
	Overall		Before December 2021		Since December 2021	
	*N* (%)	*p*	*N* (%)	*p*	*N* (%)	*p*
Immune competent	674 (47.9)	Reference	611 (50.3)	Reference	60 (32.8)	Reference
All immune suppressed	169 (52.6)	0.13	132 (64.7)	**< 0.001**	37 (32.2)	0.91
All tumors	90 (53.3)	0.19	71 (67.0)	**0.001**	19 (30.6)	0.76
Solid tumors	48 (56.5)	0.13	40 (65.6)	**0.02**	8 (34.8)	0.82
Hematologic tumors	49 (52.1)	0.43	37 (68.5)	**0.009**	12 (30.0)	0.85
All immune suppressants	87 (51.8)	0.35	60 (63.2)	**0.016**	27 (37.5)	0.48
Biological drugs^1^	34 (64.2)	0.02	25 (71.4)	**0.016**	9 (50)	0.19
Chemotherapeutics^2^	38 (55.1)	0.25	27 (71.1)	**0.013**	11 (35.5)	0.84
Corticosteroids and other drugs^2^	46 (50.0)	0.7	29 (60.4)	0.17	17 (39.5)	0.4
All connective tissue diseases	37 (66.1)	**0.008**	31 (72.1)	**0.005**	6 (46.2)	0.34
Rheumatoid arthritis	12 (63.2)	0.25	10 (76.9)	**0.091**	2 (33.3)	1
Other connective tissue diseases	25 (65.8)	**0.03**	21 (70.0)	**0.041**	4 (50.0)	0.45
Transplant recipient	16 (30.2)	**0.011**	9 (50.0)	1	7 (20.0)	0.16
Neutropenia	10 (45.5)	0.83	5 (71.4)	0.45	5 (33.3)	1
HIV/AIDS	12 (66.7)	0.15	10 (90.9)	**0.012**	2 (28.6)	1
Asplenia	4 (40.0)	0.76	4 (44.4)	0.75	1 (100.0)	0.33
Aplastic anemia	5 (55.6)	0.75	4 (57.1)	1	1 (50.0)	0.55
Other autoimmune diseases	2 (40.0)	1	1 (33.3)	0.62	1 (50.0)	0.55
A/Hypogammaglobulinemia	2 (50.0)	1	2 (66.7)	1	0 (0.0)	1

1 During the six months prior to admission.

2 During the three months prior to admission.

N: number of subjects with severe outcomes; %: percentage of subjects with severe outcomes. Significantly different *p* values (vs. immune competent) are in bold.

The proportion of severe outcomes in the two groups (immune competent and immune suppressed) was the highest at the beginning of the pandemic (>50%) and then fluctuated over time with lower proportions observed during the summer months (Supplementary Figure S4). Some high peaks observed in the immune suppressed group were likely caused by the extremely low numbers of patients (between 1 and 6) in the summer months. However, the proportion of cases with serious outcomes started to decrease steadily in February 2022 and remained around or below 20% since April 2022. The moment when a higher proportion of favorable outcomes started to be recorded was delayed by two months with respect to other identified trend changes (increase in age and co-morbidities, including immune suppression). Nonetheless, considering the two different periods identified before (end of February 2020–November 2021 and December 2021–November 2022) the overall proportion of patients with severe outcomes decreased significantly from 52.4% (743/1418) to 32.6% (97/298, *p* < 0.001).

As for the whole population, in patients with severe outcomes the proportion of immune suppressed patients among subjects hospitalized since December 2021 was significantly higher than the proportion of immune suppressed subjects among patients hospitalized before that date (Supplementary Table S2). However, a higher proportion of immune suppressed individuals was noted among patients with a severe outcome, compared to those with a favorable outcome, only when considering subjects hospitalized before December 2021 (Table 4).

**Table 4:** Immune suppressed patients among all subjects hospitalized stratified by outcome and hospitalization time.

	Immune suppressed patients		
	*N*	%	*p*
*Before December 2021*			
Patients with favorable outcomes (*N* = 675)	72	10.7	Reference
Patients with severe outcome^1^ (*N* = 743)	132	17.8	**< 0.001**
Patients who died of COVID-19 (*N* = 223)	51	22.9	**< 0.001**
*Since December 2021*			
Patients with favorable outcome (*N* = 201)	78	38.8	Reference
Patients with severe outcome^1^ (*N* = 97)	37	38.1	0.91
Patients who died of COVID-19 (*N* = 29)	13	44.8	0.55

*p* values for frequencies that are significantly different are in bold (with a significance cut-off of 0.02 due to Bonferroni correction). N: number of immune suppressed subjects; %: percentage of immune suppressed subjects.

1 Includes septic shock, intubation, ICU admission, ARDS/pneumonia, and death.

Finally, as shown in Table 3, in the period before December 2021, severe outcomes were observed significantly more frequently among immune suppressed compared to immune competent individuals both overall as well as for some sub-categories, including cancer patients, patients treated with chemotherapeutics or biological drugs, those with connective tissue diseases, and patients with HIV/AIDS. Patients with other immune suppression-associated conditions also presented elevated percentages of severe outcomes, but the statistical analyses did not evidence significant differences, likely because of the low number of patients in these groups. Nonetheless, all these differences disappeared in the period December 2021–November 2022.

#### 3.3.1. Outcome and vaccination status

Table 5 shows the outcomes in the studied sub-populations stratified by vaccination status. Compared to non-vaccinated immune competent subjects, a higher percentage of unvaccinated immune suppressed individuals experienced severe outcomes, including deaths, and longer hospitalization times. On the contrary, vaccinated patients (both immune suppressed and immune competent groups) showed a lower proportion of severe outcomes, particularly pneumonia, and the median hospitalization length among vaccinate immune competent subjects was lower. Among immune suppressed patients, vaccinated subjects experienced less severe outcomes, particularly for what concerns pneumonia and death. In the period February 2020–November 2021, a more severe outcome was still noted for both non-vaccinated groups (Supplementary Table S4), while no significant differences were observed in the period December 2021–November 2022 (Supplementary Table S5).

**Table 5:** Outcomes among immune suppressed compared to immune competent patients stratified by vaccination status.

	Immune competent					Immune suppressed						
	Non vaccinated (*N* = 1,109)		Vaccinated (*N* = 161)			Non vaccinated (*N* = 182)			Vaccinated (*N* = 107)			
Outcome												
	*N*	%	*N*	%	*p* ^1^ ^,^ ^2^	*N*	%	*p* ^1^ ^,^ ^3^	*N*	%	*p* ^1^ ^,^ ^4^	*p* ^5^
Favorable	534	48.2	119	73.9	**<0.001**	64	35.2	**0.0011**	71	66.4	**<0.001**	**<0.001**
Severe^6^	575	51.8	42	26.1		118	64.8		36	33.6		
Septic shock	20	1.8	4	2.5	0.53	1	0.5	0.34	1	0.9	1	1
Intubation	72	6.5	0	0.0	**<0.001**	6	3.3	0.13	6	5.6	0.84	0.37
ICU admission	89	8.0	5	3.1	0.023	8	4.4	0.095	8	7.5	1	0.29
Death	165	14.9	18	11.2	0.21	51	28.0	**<0.001**	10	9.3	0.12	**<0.001**
ARDS/Pneumonia	522	47.1	36	22.4	**<0.001**	102	56.0	0.025	28	26.2	**<0.001**	**<0.001**

Hospitalization length								
	Median	Median	*p* ^1^ ^,^ ^2^	Median	*p* ^1^ ^,^ ^3^	Median	*p* ^1^ ^,^ ^4^	*p* ^1^ ^,^ ^5^
	14	10	**<0.001**	16	**0.012**	14	0.33	0.25

*p* values for frequencies and medians that are significantly different are in bold. N: number of subjects; %: percentage of subjects; ICU: intensive care unit; ARDS: acute respiratory distress syndrome.

1 Significance cut-off of 0.02 due to Bonferroni correction.

2 Vaccinated immune competent vs. non-vaccinated immune competent.

3 Non-vaccinated immune suppressed vs. non-vaccinated immune competent.

4 Vaccinated immune suppressed vs. non-vaccinated immune competent.

5 Vaccinated immune suppressed vs. non-vaccinated immune suppressed.

6 Includes septic shock, intubation, ICU admission, death, and ARDS/pneumonia.

Finally, within the various categories of immune suppression that were statistically associated with severe outcomes, vaccination showed a significant beneficial effect only for cancer patients and subjects with HIV/AIDS as the proportion of patients with severe outcomes was significantly higher in the non-vaccinated groups for these patient categories (Table 6). We also attempted at assessing the effect of the vaccination in the two separate periods, but the number of patients was too small to render differences detectable (Supplementary Tables S6, S7).

**Table 6:** Patients with severe outcomes among vaccinated and non-vaccinated subjects with specific immune suppression conditions.

	Number of vaccinated (%)		Number of non-vaccinated (%)		*p*
	Severe outcome	Favorable outcome	Severe outcome	Favorable outcome	
All tumors	16 (29.6)	38 (70.4)	66 (66.7)	33 (33.3)	**< 0.001**
Biological drugs	11 (55.0)	9 (45.0)	19 (70.4)	8 (29.6)	0.36
Chemotherapeutics	12 (42.9)	16 (57.1)	23 (67.7)	11 (32.3)	0.072
Other connective tissue diseases	6 (60.0)	4 (40.0)	17 (70.8)	7 (29.2)	0.69
HIV/AIDS	1 (16.7)	5 (83.3)	11 (91.7)	1 (8.3)	**0.004**

*p* values for frequencies that are significantly different are in bold. %: percentage of subjects.

#### 3.3.2. Multivariate analyses

To evaluate the contribution of immune suppression and other factors to the severity of the outcome, a logistic regression was performed considering as potential predictors all variables identified to be associated with severe outcomes in this study (age, immunological status, vaccination, number of co-morbidities, hospitalization period, and sex). As the distribution of co-morbidities differed between patients with severe and favorable outcomes (*p* = 0.002), the number of co-morbidities was assessed as a continuous variable.

**Figure 2:**
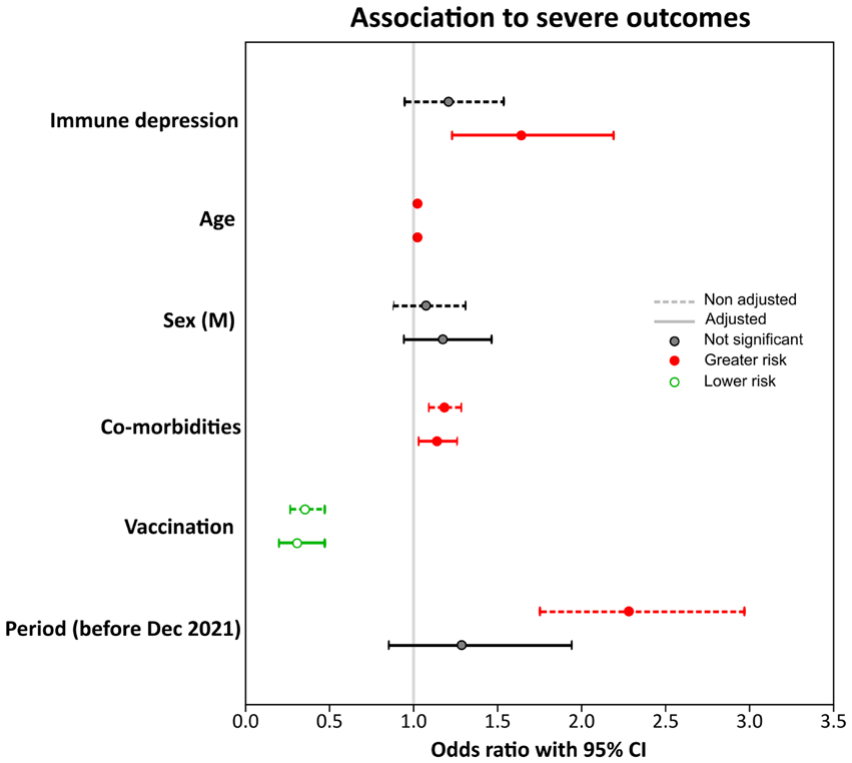
Potential predictors of severe outcomes. Odds ratios (dots) and 95% confidence intervals (lines) are indicated for each potential predictor of severe outcome when evaluated separately (simple logistic regression, dotted line) or after adjusting for the other variables (multiple logistic regression, continuous line). Results are labeled according to statistical significance as indicated in the legend. The vertical line indicates the cut-off for statistical significance.

The potential association of each variable to a severe outcome was evaluated individually as well as adjusted by all the other variables (Figure 2 and Supplementary Table S8, crude and adjusted models). While age and the presence of co-morbidities (other than immune suppression) were significantly associated with a severe outcome and vaccination resulted to have a protective effect against severe outcomes in both crude and adjusted models, immune suppression was a significant predictor of severe outcomes only after adjusting for the other variables. Immune suppression was associated with a 64% increase in the odds of severe outcome after adjusting for sex, age, number of co-morbidities, vaccination, and period of hospital admission (OR: 1.64; 95% CI: 1.23–2.19). Conversely, there was a 70% decrease in the odds of a severe outcome with vaccination (OR: 0.31; 95% IC: 0.20–0.47). The period of hospital admission, which was significantly associated with a severe outcome when analyzed as a single predictor, did not modify the protective effect of the vaccination and was not associated with an increased risk after adjusting for the other variables. This is likely due to the fact that the majority of patients during the first period were not vaccinated, while the opposite was true in the second period.

## 4. Discussion

During the three years of pandemic alert, the epidemiological and clinical features of SARS-CoV-2 kept changing while the virus was adapting to its host and spreading globally among an initially fully naïve human population that progressively acquired immunity (14). While different viral variants emerged, each characterized by different degrees of pathogenicity and transmissibility, the scientific and medical communities learned how to deal with the new disease and slowly acquired the knowledge necessary to fight it (14, 23). During this time, we collected clinical data from SARS-CoV-2-infected patients that were admitted to the COVID-19 unit of an hospital in Lombardy, the Italian region where COVID-19 initially had the heaviest impact and the first epicenter of the European epidemic (10–12). In this study, we evaluated the contribution of immune suppression to COVID-19 hospitalization and severe disease outcome to identify clinical and epidemiological trends during the three years of the SARS-CoV-2 epidemic.

### 4.1. The two different epidemiological and clinical phases of the COVID-19 epidemic

Over the whole study period, our analyses revealed the presence of two clearly different phases, which were distinguished by the different epidemiological and clinical features characterizing hospitalized subjects. During the first phase, starting at the beginning of the study (end of February 2020) and lasting until December 2021, patients were younger and had a lower number of co-morbidities, a smaller proportion of them presented immune suppression, and severe outcomes were frequent (approximately 52%). In the period that followed (December 2021–November 2022), the characteristics of the hospitalized patients changed reflecting a milder disease (subjects were older and had more co-morbidities) and the clinical picture changed with a lower proportion of severe outcomes (approximately 33%). Strikingly, the percentage of immune suppressed individuals in the second period increased dramatically, from 10–20% to 30–50%. These results may be partially influenced by the fact that, during the first few months of the epidemic, some elderly patients were not admitted to hospitals because of the heavy impact of COVID-19 on hospitals and bed scarcity. Nonetheless, although in the second period patients were older and these older patients presented significantly more immune suppression factors compared to the younger age groups, the proportion of immune suppressed subjects in the second period was significantly higher in all age groups. This rules out the possibility that the observed trend of increase in the proportion of patients with immune suppression was exclusively due to the shift in the age of the subjects.

The proportion of patients with severe outcomes was the highest at the beginning of the pandemic in both immune competent and immune suppressed groups and started to decrease around February 2022, two months after the other trends started to change. Nevertheless, a higher proportion of immune suppressed individuals among patients with severe outcomes was observed during the first period, when severe outcomes and deaths were also significantly more frequent in the groups of immune suppressed subjects. These differences disappeared in the period December 2021–November 2022.

In December 2021, Italy reached a COVID-19 first dose vaccination coverage of 80% and we can assume that, after almost 2 years of sustained SARS-CoV-2 transmission, natural immunity was also contributing to increase the strength of the immune response of the general population against the virus (22, 24). This could indicate that, from this point in time onwards, immune suppressed subjects and weaker members of the community were much more susceptible to the disease because they were not able to build an immune response strong enough to fight the infection, even after vaccination or previous exposure. Moreover, Omicron started to become the most prevalent variant in Italy around December 2021 and the shift we observed could have also been caused by the reduced pathogenicity of this viral variant (15). Indeed, while the variant Omicron is characterized by a higher transmission rate compared to previous variants, it has been shown to cause milder symptoms and to be associated with better hospital outcomes. This seems to be the case even if the vaccine effectiveness against severe illness, hospitalization, and mortality and high vaccination coverages make evaluations of its virulence more complicated (25, 26). Likewise, we could not definitely conclude whether the noted shifts were due to the reached high immunity coverage, the spread of the Omicron variant, or both.

The observed trend is nonetheless consistent with the recent decision of the World Health Organization (WHO) that COVID-19 no longer constitutes a public health emergency of international concern (PHEIC). This decision was driven by the high population-level immunity, the low virulence of the currently circulating Omicron sub-lineages, and the improved clinical case management that, all together, resulted in a decline in COVID-19-related deaths, hospitalizations, and intensive care need (27).

### 4.2. Immune suppression is associated with severe COVID-19 disease outcomes among hospitalized patients

While there are contradictory data about the association between immune suppression and severe COVID-19 outcome, possibly also due to different definitions of immune suppression in the various studies, in our population we identified a statistically significant association between them. When considering the data for the whole period, mortality was significantly higher among immune suppressed patients (20%) compared to immune competent subjects (14%). Moreover, a statistically significant increase in the frequency of severe outcomes (including mortality) was observed between the groups of immune suppressed and immune competent when we analyzed the patients of the first period alone (50% vs. 65%). Strikingly, the regression analysis showed an OR of 1.64 (1.23–2.19) for immune suppression, after adjusting for age, sex, time of hospitalization, vaccination status, and other co-morbidities.

Even though we cannot draw strong conclusions as the low number of patients in each category of immune suppression limited the power of these analyses and we could not perform a regression analysis for specific categories of immune suppression, we could observe an association between some immune suppression conditions and severe outcomes. Over the whole period, worse outcomes were noticed among patients treated with biological drugs and patients with connective tissue diseases while during the first period alone, severe outcomes were significantly more frequent also in some other categories. Finally, no differences in outcome among the various sub-populations were observed in the second period.

An association between worse outcomes among cancer patients was observed during the first period and this is consistent with published literature showing that COVID-19 is more severe in cancer patients. Additionally, previous studies have shown that hematologic cancer patients and subjects with lung cancer experience more severe COVID-19 (28, 29). In our study, we observed a worsened outcome in subjects with both hematologic and solid cancers, as well as in patients undergoing chemotherapy, and there was no difference between the two groups in terms of outcome (data not shown). Unfortunately, we could not conclude specifically about patients with lung cancer, a category particularly at high risk of severe disease (30), as this information was recorded only for a few subjects.

Patients that took biological drugs during the six months prior to hospitalization showed a worse outcome both when considering the whole period as well as when investigating the first period alone. In literature, the effect of biological drugs on COVID-19 outcomes is not entirely clear. While some studies found that immune suppressive therapies before hospitalization were not associated with in-hospital mortality, a worse outcome has been clearly documented for patients undergoing B cell-depleting therapies, including rituximab (3, 9, 31). Given the small number of patients, we could not evaluate this aspect in more detail.

While connective tissue diseases other than rheumatoid arthritis were associated with a worse outcome in our study, rheumatoid arthritis was not. As mentioned before, specific data on therapies were not considered and it is possible that we failed in identifying a correlation because worse outcomes in patients with rheumatoid arthritis seem to be therapy-dependent (i.e., rituximab and Janus kinase inhibitors) (9).

A worse outcome was observed in patients with HIV/AIDS, which were also significantly younger compared to immune competent patients. These results are in agreement with literature data (3, 6). Finally, surprisingly, we did not find more severe COVID-19 outcomes in transplant recipients, contrarily to what was observed in other studies, which recorded higher mortality in this group (3, 6). The reason for this discrepancy is not clear.

### 4.3. Considerations about natural and vaccine-mediated immunity against SARS-CoV-2

Unfortunately, data about COVID-19 vaccination were incomplete for most patients and information about the number and the dates of the received doses was available only for a few individuals. Therefore, for this study, we considered as vaccinated all patients who received at least one vaccine dose at any time prior to hospitalization. Since timing and number of doses are crucial in determining the severity of the outcome (32–34), our results may be biased as some of the vaccinated patients may not have reached a protective level of immunity at the moment of hospitalization, making the effect of vaccination less evident. Additionally, some immune depressed patients may not develop a protective response after vaccination (35, 36). Nonetheless, we observed a higher frequency of less severe outcomes, particularly for pneumonia and ARDS, in all vaccinated individuals, regardless of their level of immune competence. Additionally, the regression analysis showed a 70% decrease in the odds of a severe outcome following vaccination, after adjusting for the other investigated variables. These results confirm that vaccination has a strong protective effect, also on immune suppressed individuals. Vaccination was also associated with a reduction of COVID-19-related fatalities among immune suppressed subjects. Interestingly, vaccination reduced significantly the severity of the outcome specifically for oncologic patients and subjects with HIV/AIDS, as also previously reported (37, 38).

While non-vaccinated immune suppressed patients were hospitalized for longer periods compared to non-vaccinated immune competent subjects, vaccination reduced hospitalization times in the immune competent group. However, as information about hospitalization in other facilities for transferred patients could not be retrieved, this data must be interpreted with caution as, especially at the beginning of the emergence, patients were transferred frequently between facilities.

We also documented a positive effect of the vaccination when analyzing the first period separately, although it was of weaker intensity. Nonetheless, we need to consider that during the first period, only a small proportion of patients was vaccinated (4.4%), limiting the power of the analysis. On the other hand, no strong effect due to the vaccination was detected during the second period but, during later stages of the epidemic, many of the non-vaccinated subjects may have had naturally acquired immunity against SARS-CoV-2. The impossibility of controlling for previous infections (no data on antibody levels were available for this investigation) made it impossible to discriminate between first infections and reinfections and the frequency of reinfections was likely higher in the second period. A high level of background natural immunity would make outcome measurements in non-vaccinated and vaccinated groups similar since previous immunity is effective in protecting against severe forms of COVID-19 (32). Therefore, we postulate that the lack of differences in outcomes between vaccinated and non-vaccinated patients during the second period was due to a high percentage of non-vaccinated subjects possessing naturally acquired antibodies against SARS-CoV-2.

Finally, we could not properly evaluate vaccine-related disease outcomes in sub-groups of subjects with different types of immune suppression in the two periods. This is due to very low numbers of vaccinated subjects in the first period and of non-vaccinated subjects in the second period, impeding a meaningful assessment. One also needs to consider that only 17% of the subjects included in this study were vaccinated and these were mostly hospitalized during the second period.

In any case, in regression analyses, vaccination was always protective against severe outcomes, independently of whether the period of hospital admission was included or not in the model (OR of approximately 0.3 with upper bound CI < 0.5). On the other hand, the period of hospitalization was a significant predictor of severe outcomes only when included in a model that did not consider vaccination status. This suggests that the differences in outcome between the two periods can be explained by the different vaccination status of the two sub-populations.

### 4.4. Conclusion

Despite some limitations, including the relatively low number of patients in some sub-populations (particularly for what regards specific immune suppression conditions and vaccinated and non-vaccinated subjects during the first and second period, respectively) and the unavailability of some important data, such as details on times and doses of vaccination or specific information for certain types of immune suppression, this study has the strength of including data collected from patients hospitalized since the very beginning of the COVID-19 hospitalization insurgence. This allowed us to detect shifts in epidemiological and clinical characteristics of hospitalized patients throughout almost three years and draw conclusions about the clinical significance of immune suppression during the various stages of the epidemic.

During the first part of the COVID-19 epidemic, hospitalized patients were younger, had fewer co-morbidities and a lower proportion of them had factors of immune suppression. After adjusting for other factors, immune suppression was responsible for an overall 64% increase in the odds of severe outcomes and different conditions seemed to contribute differently to the severity of the outcome. While the spread of the less pathogenic Omicron variant may have been partly responsible for the reduced severity of the disease, a higher level of (natural and vaccine-induced) immunity in the general population significantly contributed to the observed shift in the characteristics of the hospitalized patients. Metanalyses or studies with a larger number of patients will be required to draw stronger conclusions for specific categories of immune suppression and determine their influence on COVID-19-related hospitalizations and severe outcomes throughout the various stages of the epidemic. Nonetheless, our results show that immune suppression is still a relevant co-morbidity in the clinical course of COVID-19 patients.

## Data availability statement

The datasets presented in this article are not readily available due to the nature of the research, because of ethical reasons and of the sensitive nature of the research data, supporting data is not available. Requests to access the datasets should be directed to AB, alessandra.bandera@policlinico.mi.it.

## Ethics statement

The studies involving humans were approved by the Medical Ethics Committee of the Fondazione IRCCS Ca′ Granda Ospedale Maggiore Policlinico (EC approval 241_2020, 17 March 2020). The studies were conducted in accordance with the local legislation and institutional requirements. The participants provided their written informed consent to participate in this study.

## Author contributions

MC: Conceptualization, Data curation, Formal analysis, Investigation, Methodology, Project administration, Visualization, Writing – original draft. MM: Supervision, Writing – review & editing. CB: Data curation, Resources, Writing – review & editing. AM: Data curation, Resources, Writing – review & editing. TM: Data curation, Validation, Writing – review & editing. SB: Validation, Writing – review & editing. FB: Data curation, Resources, Writing – review & editing. CC: Data curation, Resources, Writing – review & editing. GC: Data curation, Resources, Writing – review & editing. AN: Data curation, Resources, Writing – review & editing. FP: Data curation, Resources, Writing – review & editing. MT: Data curation, Resources, Writing – review & editing. SV: Funding acquisition, Writing – review & editing. SA: Data curation, Resources, Writing – review & editing. MR: Funding acquisition, Writing – review & editing. AG: Data curation, Funding acquisition, Resources, Writing – review & editing. AB: Data curation, Funding acquisition, Project administration, Resources, Supervision, Writing – review & editing.

## COVID-19 Network Study Group

Silvano Bosari, Luigia Scudeller, Giuliana Fusetti, Laura Rusconi, Silvia Dell’Orto, Daniele Prati, Luca Valenti, Silvia Giovannelli, Maria Manunta, Giuseppe Lamorte, Francesca Ferarri, Davide Mangioni, Laura Alagna, Giorgio Bozzi, Andrea Lombardi, Riccardo Ungaro, Giuseppe Ancona, Gianluca Zuglian, Matteo Bolis, Nathalie Iannotti, Serena Ludovisi, Agnese Comelli, Giulia Renisi, Simona Biscarini, Valeria Castelli, Emanuele Palomba, Marco Fava, Valeria Fortina, Arianna Liparoti, Andrea Pastena, Carlo Alberto Peri, Paola Saltini, Giulia Viero, Teresa Itri, Valentina Ferroni, Valeria Pastore, Roberta Massafra, Maria Teresa Curri, Alice Rizzo, Stefano Scarpa, Alessandro Giommi, Rosaria Bianco, Grazia Eliana Chitani, Roberta Gualtierotti, Barbara Ferrari, Raffaella Rossio, Nadia Boasi, Erica Pagliaro, Costanza Massimo, Michele De Caro, Andrea Giachi, Nicola Montano, Barbara Vigone, Chiara Bellocchi, Angelica Carandina, Elisa Fiorelli, Valerie Melli, Eleonora Tobaldini, Maura Spotti, Leonardo Terranova, Sofia Misuraca, Alice D’Adda, Silvia Della Fiore, Marta Di Pasquale, Marco Mantero, Martina Contarini, Margherita Ori, Letizia Morlacchi, Valeria Rossetti, Andrea Gramegna, Maria Pappalettera, Mirta Cavallini, Agata Buscemi, Marco Vicenzi, Irena Rota, Monica Solbiati, Ludovico Furlan, Marta Mancarella, Giulia Colombo, Giorgio Colombo, Alice Fanin, Mariele Passarella, Valter Monzani, Angelo Rovellini, Laura Barbetta, Filippo Billi, Christian Folli, Silvia Accordino, Diletta Maira, Cinzia Maria Hu, Irene Motta, Natalia Scaramellini, Anna Ludovica Fracanzani, Rosa Lombardi, Annalisa Cespiati, Matteo Cesari, Tiziano Lucchi, Marco Proietti, Laura Calcaterra, Clara Mandelli, Carlotta Coppola, Arturo Cerizza; Intensive Care Unit: Antonio Maria Pesenti, Giacomo Grasselli, Alessandro Galazzi (Fondazione IRCCS Ca′ Granda Ospedale Maggiore Policlinico); Igor Monti, Alessia Antonella Galbusera (Istituto di Ricerche Farmacologiche Mario Negri IRCCS).

## Funding

The author(s) declare financial support was received for the research, authorship, and/or publication of this article. This research was funded by the PREP-COVID project financed by Bolton Hope Foundation and the Fondazione Cariplo 2021-4236 LLC Network project. SB is funded by the European Union’s Horizon programme under grant agreement no. 101046314 (END-VOC project).

## Conflict of interest

The authors declare that the research was conducted in the absence of any commercial or financial relationships that could be construed as a potential conflict of interest.

The author(s) declared that they were an editorial board member of Frontiers, at the time of submission. This had no impact on the peer review process and the final decision.

## Publisher’s note

All claims expressed in this article are solely those of the authors and do not necessarily represent those of their affiliated organizations, or those of the publisher, the editors and the reviewers. Any product that may be evaluated in this article, or claim that may be made by its manufacturer, is not guaranteed or endorsed by the publisher.
