# Supplementary material for: The role of immune suppression in COVID-19 hospitalization: clinical and epidemiological trends over three years of SARS-CoV-2 epidemic
Source: Front Med (Lausanne). 2023 Sep 7;10:1260950. doi: 10.3389/fmed.2023.1260950 (PMC10513414; doi:10.3389/fmed.2023.1260950)
Supplement: Supplementary file 1 [file Data_Sheet_1.PDF]

Supplementary Material

The role of immune suppression in COVID-19 hospitalization: clinical and epidemiological trends over three years of SARS-CoV-2 epidemic

Marta Canuti*, Maria Cristina Monti, Chiara Bobbio, Antonio Muscatello, Toussaint Muheberimana, Sante Leandro Baldi, Francesco Blasi, Ciro Canetta, Giorgio Costantino, Alessandro Nobili, Flora Peyvandi, Mauro Tettamanti, Simone Villa, Stefano Aliberti, Mario C. Raviglione, Andrea Gori, Alessandra Bandera*, COVID-19 Network Study Group

*** Correspondence:** MC: marta.canuti@gmail.com, AB: alessandra.bandera@policlinico.mi.it

Supplementary Table S1. Differences between the frequencies of females, vaccinated subjects, and co-morbidities, and between the median ages of immune suppressed patients compared to the immune competent group.

Group	Frequency (percentage)										Median
		Co-morbidities									
	**F**	**Resp**	**Card**	**Neph**	**GI/Li**	**Met**	**Neur**	**No**	**>1**	**V**	**Age**
Immune competent (N=1406)	32.5	13.2	51.3	7.7	14.0	35.8	30.2	30.2	40.3	12.7	64
All tumors (N=169)	**42.6**	16.6	56.8	**12.4**	**27.2**	33.1	9.5	**19.5**	46.7	**35.3**	**72.0**
Solid tumors (N=85)	**44.7**	18.8	61.2	5.9	9.4	41.2	11.8	22.4	48.2	**27.0**	**73.0**
Hematologic tumors (N=94)	39.4	14.9	55.3	**19.1**	**39.4**	**24.5**	9.6	**17.0**	45.7	**40.2**	**71.5**
All immune suppressants (N=168)	**47.6**	**19.0**	51.8	**17.3**	**33.3**	36.3	8.3	**19.0**	**50.0**	**44.1**	66.0
Biological drugs (N=53)[1]	**54.7**	**54.7**	45.3	13.2	**26.4**	28.3	7.5	24.5	47.2	**42.6**	64.0
Chemotherapeutics (N=69)[2]	43.5	15.9	52.2	11.6	**31.9**	31.9	13.0	**17.4**	47.8	**45.2**	**72.0**
Corticosteroids and other drugs (N=92)[2]	**47.8**	**21.7**	53.3	**23.9**	**37.0**	41.3	8.7	**18.5**	**59.8**	**45.9**	63.5
Connective tissue diseases (N=56)	**51.8**	21.4	62.5	**17.9**	16.1	48.2	14.3	**16.1**	**60.7**	**34.0**	67.5
Rheumatoid arthritis (N=19)	42.1	21.1	63.2	21.1	10.5	52.6	21.1	15.8	**68.4**	**47.1**	69.0
Other connective tissue diseases (N=38)	**55.3**	21.1	60.5	15.8	18.4	44.7	10.5	18.4	55.3	**29.4**	67.0
Transplant recipient (N=53)	39.6	20.8	**66.0**	**43.4**	**58.5**	**52.8**	1.9	**9.4**	**75.5**	**69.4**	**57.0**
Neutropenia (N=22)	40.9	22.7	59.1	18.2	**59.1**	40.9	13.6	**4.5**	**68.2**	**61.9**	68.5
HIV/AIDS (N=18)	27.8	5.6	**22.2**	0.0	**38.9**	16.7	44.4	44.4	22.2	**33.3**	**56.0**
Asplenia (N=10)	50.0	0.0	50.0	10.0	**40.0**	30.0	10.0	10.0	30.0	10.0	62.0
Aplastic anemia (N=9)	44.4	0.0	66.7	33.3	**55.6**	22.2	11.1	11.1	55.6	22.2	71.0
Other autoimmune diseases (N=5)	66.7	0.0	20.0	0.0	0.0	20.0	0.0	60.0	20.0	20.0	71.0
A/Hypogammaglobulinemia (N=4)	50.0	25.0	0.0	0.0	25.0	0.0	0.0	50.0	0.0	12.5	**41.0**

Frequencies and medians that are significantly different compared to those of the group of immune competent patients are in bold. F: female; Resp: respiratory diseases; Card: cardiovascular diseases; Neph: nephropathies; GI/Li: gastrointestinal/liver diseases; Met: metabolic diseases; Neur: neurologic diseases; No: no comorbidities; >1: co-morbidities of more than 1 category; V: vaccinated.

[1]during the six months prior to admission; [2]during the three months prior to admission

Supplementary Table S2. Immune suppressed patients among hospitalized patients stratified by hospitalization period, age, and outcome.

Before December 2021	N	%	Since December 2021	N	%	p
**All outcomes**						
Total (N=1418)	204	14.4	Total (N=298)	115	38.6	< **0.001**
18-50 (N=290)	30	10.3	18-50 (N=43)	17	39.5	< **0.001**
51-70 (N=610)	86	14.1	51-70 (N=110)	47	42.7	< **0.001**
>70 (N=517)	88	17.0	>70 (N=145)	51	35.2	< **0.001**
**Severe outcome**						
Total (N=743)	132	17.7	Total (N=97)	37	38.1	< **0.001**
18-50 (N=109)	13	11.9	18-50 (N=13)	5	38.5	**0.024**
51-70 (N=299)	58	19.4	51-70 (N=39)	15	38.5	**0.012**
>70 (N=334)	61	18.3	>70 (N=45)	17	37.8	**0.005**
**Death**						
Total (N=223)	51	22.9	Total (N=29)	13	44.8	**0.021**
18-50 (N=5)	2	40.0	18-50 (N=1)	1	100.0	1
51-70 (N=43)	18	41.9	51-70 (N=6)	4	66.7	0.39
>70 (N=175)	31	17.7	>70 (N=22)	8	36.4	**0.039**

P values for statistically significant differences between the two periods are highlighted in bold. N: number of immune suppressed subjects; %: percentage of immune suppressed subjects

Supplementary Table S3. Patients with co-morbidities in more than one considered category during the two time periods February 2020-November 2021 and December 2021-November 2022.

Before December 2021 (N=1418)	N	%	Since December 2021 (N=298)	N	%	p
Without immune suppression	578	34.4	Without immune suppression	148	49.7	**0.005**
Including immune suppression	632	44.6	Including immune suppression	183	61.4	< **0.001**

N and %: number and percentage of subjects with co-morbidities in more than 1 category including and excluding immune suppression as co-morbidity category. P values for statistically significant differences between the two periods are highlighted in bold

Supplementary Table S4. Outcomes among immune suppressed compared to immune competent patients stratified by vaccination status in the period between the end of February 2020 and November 2021.

	**Immune competent**					**Immune suppressed**						
	**Non vaccinated (N=1057)**		**Vaccinated (N=41)**			**Non vaccinated (N=171)**			**Vaccinated (N=16)**			
**Outcome**												
	**N**	**%**	**N**	**%**	**$P^{1,2}$**	**N**	**%**	**$P^{1,3}$**	**N**	**%**	**$P^{1,4}$**	**P^{5}**
**Favorable**	506	47.9	32	78.0	**< 0.001**	58	33.9	**< 0.001**	7	43.8	0.81	0.42
**Severe[6]**	551	52.1	9	22.0		113	66.1		9	56.3		
**Septic shock**	19	1.8	0	0.0	1	1	0.6	0.34	0	0.0	1	1
**Intubation**	71	6.7	0	0.0	0.11	6	3.5	0.11	3	18.8	0.092	**0.032**
**ICU admission**	88	8.3	0	0.0	0.070	8	4.7	0.12	3	18.8	0.15	0.056
**Death**	161	15.2	5	12.2	0.82	48	28.1	**< 0.001**	2	12.5	1	0.24
**ARDS/Pneumonia**	500	47.3	8	19.5	**< 0.001**	98	57.3	**0.015**	6	37.5	0.46	0.19
**Hospitalization length**												
	**Median**		**Median**		**p**	**Median**		**p**	**Median**		**p**	**p**
	14		9		0.055	16		**0.017**	15.5		0.50	0.89

P values for frequencies and medians that are significantly different are in bold. N: number of subjects; %: percentage subjects; ICU: intensive care unit; ARDS: acute respiratory distress syndrome.

[1]significance cut-off of 0.02 due to Bonferroni correction; [2]vaccinated immune competent vs. non-vaccinated immune competent; [3]non-vaccinated immune suppressed vs. non-vaccinated immune competent; [4]vaccinated immune suppressed vs. non-vaccinated immune competent; [5]vaccinated immune suppressed vs. non-vaccinated immune suppressed; [6]includes septic shock, intubation, ICU admission, death, and ARDS/pneumonia

Supplementary Table S5. Outcomes among immune suppressed compared to immune competent patients stratified by vaccination status in the period between December 2021 and November 2022.

	**Immune competent**					**Immune suppressed**						
	**Non vaccinated (N=49)**		**Vaccinated (N=99)**			**Non vaccinated (N=10)**			**Vaccinated (N=79)**			
**Outcome**												
	**N**	**%**	**N**	**%**	**P[1,2]**	**N**	**%**	**P[1,3]**	**N**	**%**	**P[1,4]**	**P[5]**
**Favorable**	26	53.1	68	68.7	0.063	5	50.0	1	53	67.1	0.14	0.31
**Severe[6]**	23	46.9	31	31.3		5	50.0		26	32.9		
**Septic shock**	1	2.0	3	3.0	1	0	0.0	1	1	1.3	1	1
**Intubation**	1	2.0	0	0.0	0.33	0	0.0	1	4	5.1	0.65	1
**ICU admission**	1	2.0	5	5.1	0.66	0	0.0	1	5	6.3	0.41	1
**Death**	3	6.1	11	11.1	0.39	3	30.0	0.055	8	10.1	0.53	0.11
**ARDS/Pneumonia**	21	42.9	27	27.3	0.062	4	40.0	1	21	26.6	0.057	0.46
**Hospitalization length**												
	**Median**		**Median**		**p**	**Median**		**p**	**Median**		**p**	**p**
	14		10		0.027	19		0.3	14		0.79	0.31

P values for frequencies and medians that are significantly different are in bold. N: number of subjects; %: percentage of subjects; ICU: intensive care unit; ARDS: acute respiratory distress syndrome.

[1]significance cut-off of 0.02 due to Bonferroni correction; [2]vaccinated immune competent vs. non-vaccinated immune competent; [3]non-vaccinated immune suppressed vs. non-vaccinated immune competent; [4]vaccinated immune suppressed vs. non-vaccinated immune competent; [5]vaccinated immune suppressed vs. non-vaccinated immune suppressed; [6]includes septic shock, intubation, ICU admission, death, and ARDS/pneumonia

Supplementary Table S6. Patients with severe outcomes among vaccinated and non-vaccinated subjects with specific immune suppression conditions in the period between the end of February 2020 and November 2021.

	Number of vaccinated (%)		Number of non-vaccinated (%)		p
	Severe outcome	Favorable outcome	Severe outcome	Favorable outcome	
**All tumors**	2 (40.0)	3 (60.0)	62 (68.1)	29 (31.9)	0.33
**Biological Drugs**	4 (66.7)	2 (33.3)	18 (72.0)	7 (28.0)	1
**Chemotherapeutics**	3 (100)	0 (0.0)	21 (70.0)	9 (30.0)	0.55
**Other connective tissue diseases**	2 (66.7)	1 (33.3)	17 (70.8)	7 (29.2)	1
**HIV/AIDS**	0 (0.0)	0 (0.0)	10 (90.9)	1 (9.1)	/

A / indicated that it was not possible to calculate the p-value. %: percentage of subjects

Supplementary Table S7. Patients with severe outcomes among vaccinated and non-vaccinated subjects with specific immune suppression conditions in the period between December 2021 and November 2022.

	Number of vaccinated (%)		Number of non-vaccinated (%)		p
	Severe outcome	Favorable outcome	Severe outcome	Favorable outcome	
**All tumors**	14 (28.6)	35 (71.4)	4 (50.0)	4 (50.0)	0.25
**Biological Drugs**	7 (50.0)	7 (50.0)	1 (50.0)	1 (50.0)	1
**Chemotherapeutics**	9 (36.0)	16 (64.0)	2 (50.0)	2 (50.0)	0.62
**Other connective tissue diseases**	4 (57.1)	3 (42.9)	0 (0.0)	0 (0.0)	/
**HIV/AIDS**	1 (16.7)	5 (83.3)	1 (100.0)	0 (0.0)	0.29

A / indicated that it was not possible to calculate the p-value. %: percentage of subjects

Supplementary Table S8. Potential predictors of severe outcomes evaluated separately (simple logistic regression) or after adjusting for the other variables (multiple logistic regression).

	**Simple logistic regression**			**Multiple logistic regression**		
	**OR**	**95% CI**	**p**	**OR**	**95% CI**	**p**
**Immune suppression**	1.21	0.95-1.54	0.13	1.64	1.23-2.19	**<0.001**
**Age**	1.02	1.02-1.03	**<0.001**	1.02	1.02-1.03	**<0.001**
**Sex (male)**	1.07	0.88-1.31	0.48	1.43	0.94-1.46	0.14
**Co-morbidities**[1]	1.18	1.09-1.28	**<0.001**	1.14	1.03-1.26	**0.011**
**Vaccination**	0.35	0.27-0.47	**<0.001**	0.31	0.20-0.47	**<0.001**
**Period (before Dec 2021)**	2.28	1.75-2.97	**<0.001**	1.29	0.85-1.94	0.23

OR: odds ratio; CI: confidence interval

[1]1, 2, 3, >3 included as continuous variables

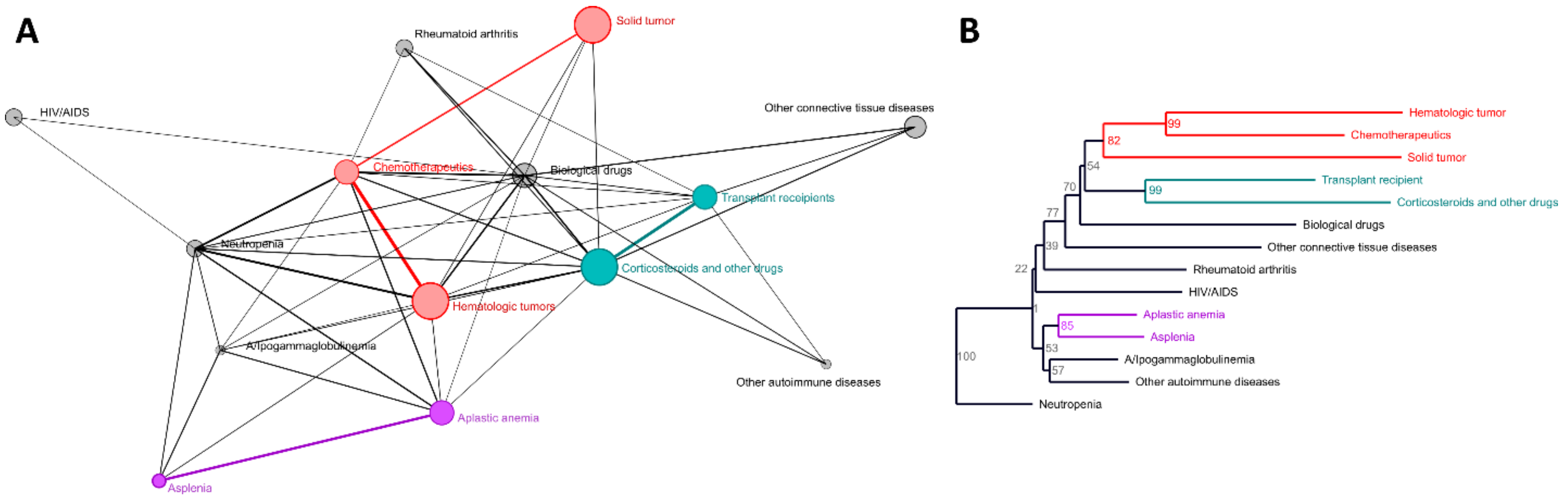

Supplementary Figure S1. Correlation between the various factors associated with immune suppression investigated in this study. In the network analysis (**A**) the size of each node is proportional to the number of patients having that condition, and the lines' thickness proportionally represents the association strength. In the clustering analysis (**B**) branch support (bootstrap) is indicated at basal nodes. Strong connections identified by both methods are highlighted with the same color.

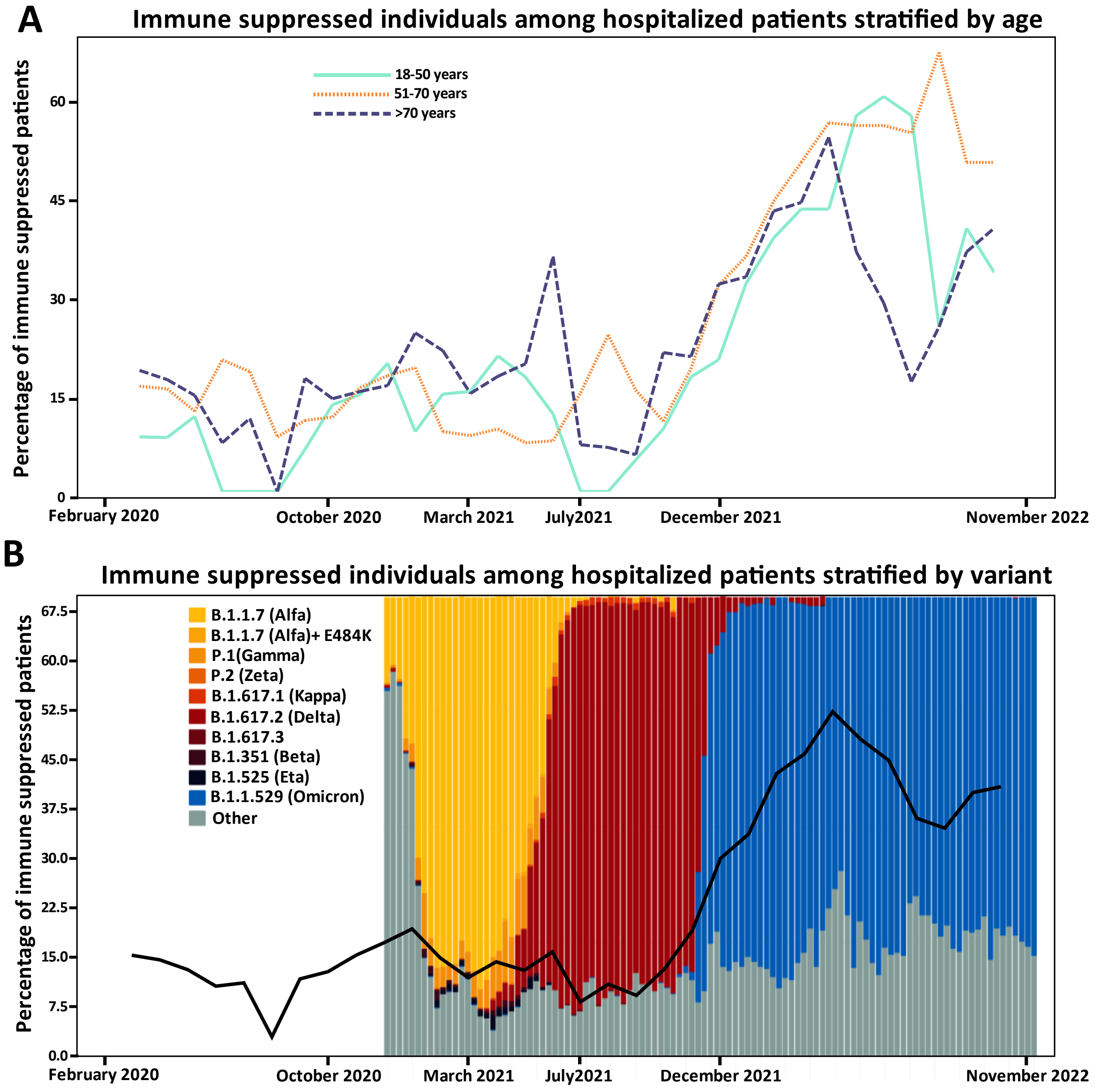

Supplementary Figure S2. Immune suppressed patients stratified by age (A) and variant (B) over time. The graphs show the proportion of immune suppressed subjects among hospitalized patients (stratified by age in A and overall in B) over time and each timepoint includes the data of 3 months – indicated timepoint +/- 1 month. In panel B, the relative frequency of variants circulating in Italy in the same period is shown as colored bars in the background (image reproduced from (1)) with each variant labeled with a different color, as indicated by the legend.

1. Istituto Superiore di Sanita'. Prevalenza e distribuzione delle varianti di SARS-CoV-2 di interesse per la sanità pubblica in Italia. Rapporto n. 28 del 3 febbraio 2023 (dati aggiornati al 30 gennaio 2023). (2023) https://www.epicentro.iss.it/

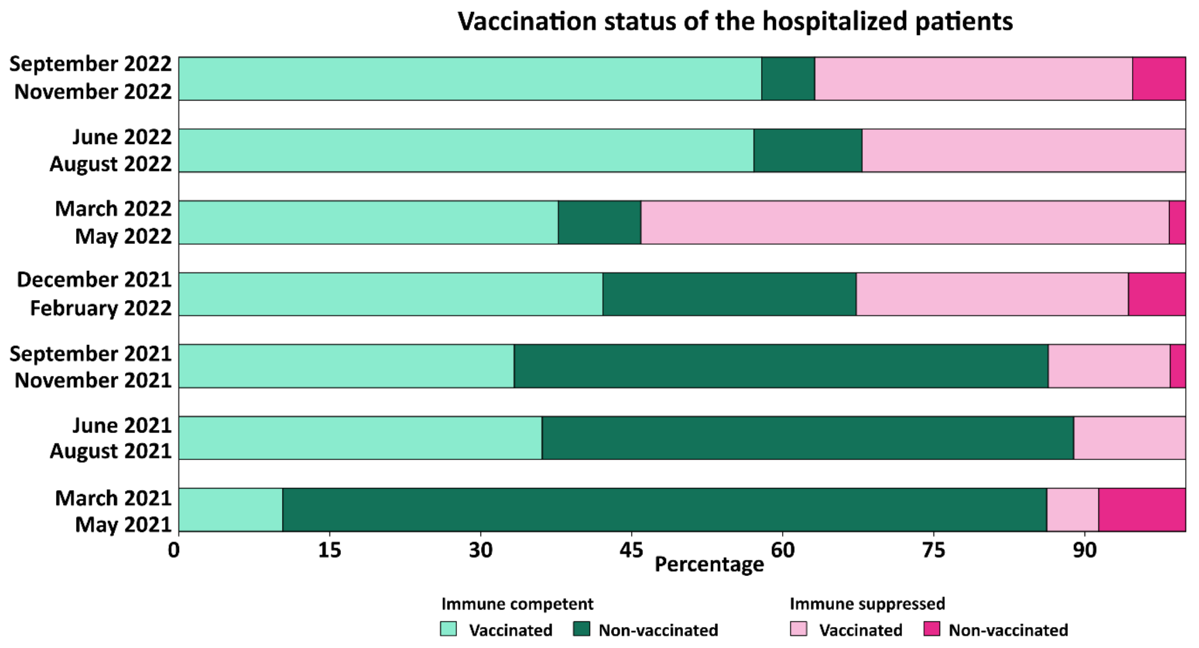

Supplementary Figure S3. Vaccination status of the hospitalized patients. Relative frequencies of vaccinated and non-vaccinated subjects according to their immune status are shown for three-month periods from the beginning of vaccination administration in Italy (March 2021) until the end of the study.

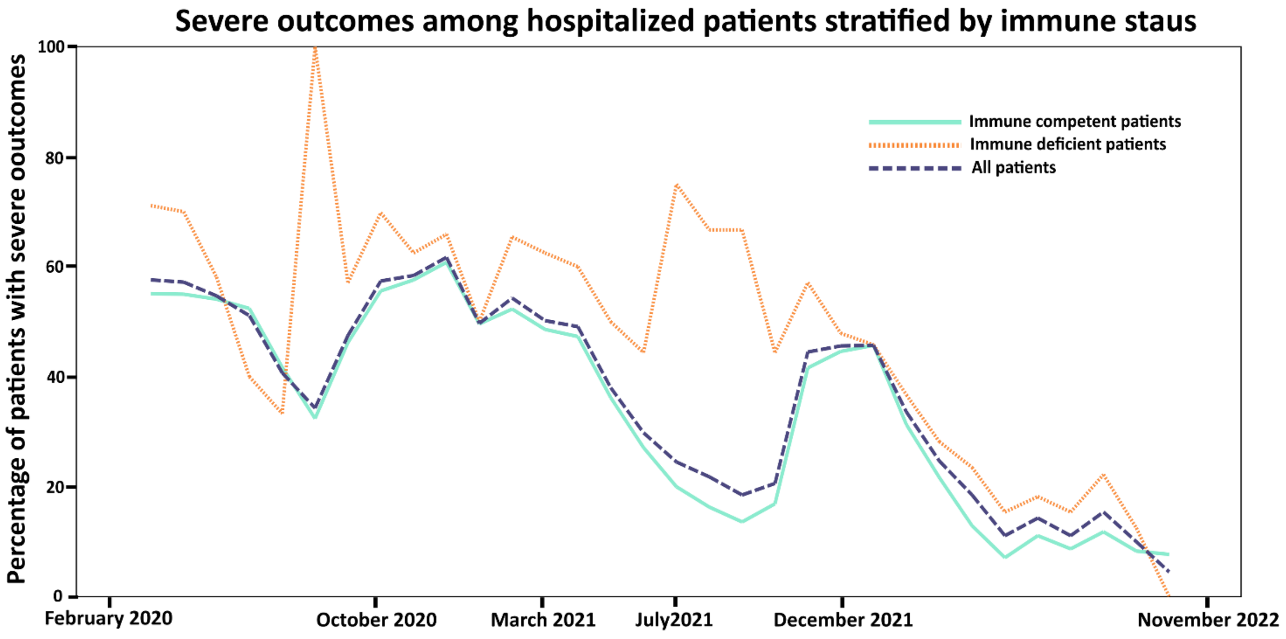

Supplementary Figure S4. Severe outcomes among hospitalized patients stratified by immune status. The graph shows the proportion of patients with severe outcomes among hospitalized patients stratified by immune status. Each time point includes data of 3 months – the indicated timepoint +/- 1 month.
